# Enhanced Diffuse Reflectance and Microstructure Properties of Hybrid Titanium Dioxide Nanocomposite Coating

**DOI:** 10.1186/s11671-018-2763-3

**Published:** 2018-10-22

**Authors:** Hai Lu, Meng Huang, Ke-Sheng Shen, Jun Zhang, Shi-Qiang Xia, Chao Dong, Zong-Gang Xiong, Ting Zhu, Da-Peng Wu, Bo Zhang, Xian-Zhou Zhang

**Affiliations:** 1 Engineering Laboratory for Optoelectronic Technology and Advanced Manufacturing, Henan Normal University, Xinxiang, 453007 China; 2 School of Chemistry and Chemical Engineering, Henan Normal University, Xinxiang, 453007 Henan China; 3 Xinxiang Baihe O.E. Co., Ltd, Xinxiang, 453731 China

**Keywords:** Optical properties, Microstructure, TiO_2_ nanocrystals, Coating

## Abstract

In this research, we studied enhanced diffuse reflectance that can be achieved by excitations of multiple-scattering in a hybrid micro-structured titanium dioxide coating. Conventional approaches to obtain diffuse reflection structure rely heavily on exciting the scattering of randomly textured surface, whereas here, we reveal numerically and experimentally that, besides interface scattering, bulk scattering of ordered-disordered hybrid structure can be also employed to obtain highly efficient diffuse reflector. The diffuse reflectance over the measured wavelength region increases significantly with thickness, while angle and polarization-dependent specular reflections are suppressed. These results show the potential to be used as a highly efficient diffuse reflector or for applications in various advanced fields of photonics related to light extractions and diffusers.

## Background

Rough-surfaces-induced light scattering responses, especially diffuse reflection, serve as the cornerstone of many branches of optics and materials science [1–3] and play a central role in lots of exotic optical and photonic phenomena [4–7]. Besides the relatively intuitional surface scattering of randomly textured dielectric interfaces [8, 9], it was recently discovered that bulk scattering exists within inhomogeneous structure, which stems from cross-correlation parameters between roughnesses or inhomogeneities [10, 11]. Consequently, a new branch of diffuse reflector emerges, which relies on a full exploitation of excitations and interferences of both surface and bulk scattering [12, 13] and enables much more flexible control of both magnitudes and polarizations of the electromagnetic fields [14, 15]. Moreover, such a field rapidly hybridizes with other branches of plasmonics, optical nanoantennas, and metamaterials, which renders enormous extra freedom for manipulations of various kinds of light-matter interactions and makes possible lots of novel photonic functionalities and devices [16–18].

A recent rather remarkable achievement based on the microstructure diffuse reflector is light management realized in various optical components [19–21]. When the light is reflected back from the diffuse reflector at the rear side, escaped light can be effectively eliminated at the front surface due to the transverse wave vector of the scattering light beyond the light cone of air. This is of great importance for various applications including solar cell, illumination, and many other applications related to light-matter interaction enhancement in devices [22–24]. Nevertheless, similar to many novel functionalities obtained in surface-relief structures and nanoparticle-based structures [16–24], the existing approaches to obtain diffuse reflector heavily rely on the excitations of the scattering of randomly textured surface [14, 15]. Then it is vital to ask: Can the diffuse reflectors be supported by interface and bulk scattering simultaneously to realize better functionalities?

Here in this paper, we report new observations of enhanced diffuse reflection in one platform by patterned ellipsoidal TiO_2_ nanoparticle assemblies. Firstly, we fabricated different hybrid structures and analyzed their diffuse reflection spectrum. It is revealed that hybrid microstructure coating composed of TiO_2_ particles-based three-dimensional spheres can totally substitute for non-absorbing powder, such as ultrahigh-purity fumed silica [23], to obtain highly efficient diffuse reflectors. And then, we performed finite difference time domain (FDTD) simulations to investigate this hybrid microstructure coating for diffuse reflection, as well as for bulk scattering. In addition, we also show that specular reflection of this hybrid microstructure coating can be greatly suppressed to achieve isotropic scattering.

## Methods

### Preparation of TiO_2_ Products

Tetrabutyl titanate (12.5 mL) was slowly added into a mixture solution of 50 mL hydrogen peroxide (H_2_O_2_, 30 wt%) and 5 mL ammonia (NH_4_OH, 26–28 wt%) dropwise in a 500 mL beaker with continuous shaking. Afterwards, cold distilled water was poured into the beaker to yield a saffron yellow precursor solution with a final volume of 200 mL. The precursor solution was filtered to remove the undissolved yellow bulks occasionally floating on the solution. Then, 10 mL of this yellow precursor was extracted and transferred into a 50 mL Teflon container with additions of 10 mL distilled water and 20 mL absolute ethanol. The mixture was tightly sealed with a stainless jacket and heated at 180 °C for 10 h. The final residue was centrifuged and washed with water and ethanol, respectively. Finally, the as-prepared sample was dried at 60 °C for 2 h. In addition, the precursor dosage was adjusted to 5 mL to prepare the anatase TiO_2_ nanocrystals.

### Fabrication of Hybrid TiO_2_ Nanocomposite Coating

The hybrid TiO_2_ nanocomposite coatings are grown through utilizing self-made anatase TiO_2_ nanocrystal deposited on a fluorine-doped tin oxide glass substrate. The fabrication method consists of three steps. First, self-made anatase TiO_2_ nanocrystals and its assemblies were selectively prepared via a solvothermal method by altering the peroxotitanium complex precursor dosage. And then, these nanocrystals or assemblies were spread onto the substrate by doctor-blade method with adhesive tape to control the coating thickness. At last, after dried in air, the coating was heated up to 450 °C at a rate of 5 °C/min and maintained for 30 min.

### Characterization

The structures of the fabricated coatings were characterized by field-emission scanning electron microscopy (HITACHI S4800). And the structural details of these assemblies can be obtained by transmission electron microscopy (Tecnai F30). The XRD pattern of the coatings were tested by Rigaku D/max-2500 diffractometer with Cu Kα radiation, λ = 0.1542 nm, 40 kV, 100 mA. The diffuse reflectance and the polarization-dependent specular reflectance of the coatings were measured, respectively, using a spectrophotometer (Angilent Carry 5000) equipped with 110 mm integrating sphere and variable angle specular reflectance accessory.

## Results and Discussion

### The Diffuse Reflectance Properties of Four Types of Microstructured TiO_2_ Coatings

**Fig. 1:**
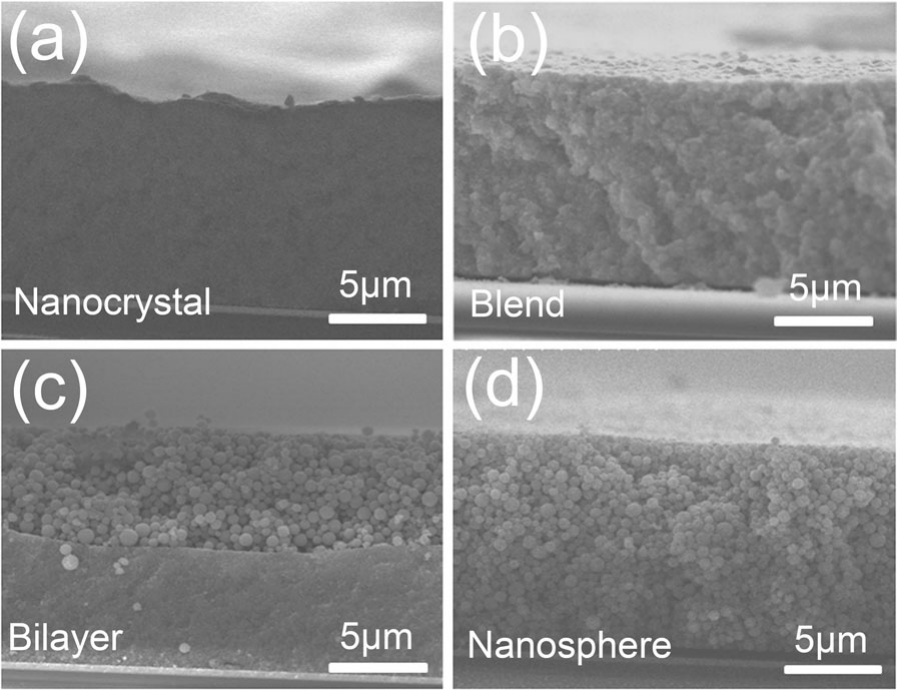
The SEM images of microstructured coatings: **a** nanocrystal coating, **b** blend coating, **c** bilayer coating, and **d** nanosphere coating. The thickness of coatings is all limited at ~ 14 μm

Here, we have fabricated four types of microstructured coating as shown in Fig. 1. They are pure nanocrystal coating, blend and bilayer coating with ellipsoidal nanocrystal and spheroidal assembly, and pure spheroidal assembly coating, respectively, and labeled as nanocrystal, blend, bilayer, and nanosphere. It should be noted that the process differences which lead to these coating structures mainly come from the different coating materials and the order of preparation. The pure nanocrystal and spheroidal assembly coatings are made by TiO_2_ nanocrystals and spheroidal assemblies, respectively. But for the blend coating, the ellipsoidal nanocrystals and spheroidal assemblies are equally mixed in weight. The bilayer coating was constructed by the doctor-blade method through two-step calcination as stated in “Fabrication of Hybrid TiO_2_ Nanocomposite Coating” section. Firstly, nanocrystal slurry was spread onto substrate. And then, after calcination, another layer of spheroidal assembly slurry was deposited on the semi-transparent layer and annealed with the same heating profile. The structures of the four fabricated coatings are characterized by field-emission scanning electron microscopy as shown in Fig. 1a–d. The thicknesses of coatings are all limited to 14 μm, and the bilayer coating is composed of ellipsoidal nanocrystal layer and spheroidal assembly layer equal in thickness (~ 7 μm). As the TiO_2_ nanocrystals grow with different sizes, they ultimately assemble to produce different diameters of the sphere. In Fig. 1, the obtained sizes in ellipsoidal TiO_2_ nanocrystal and spheroidal assembly are about 20 and 100 nm, respectively.

**Fig. 2:**
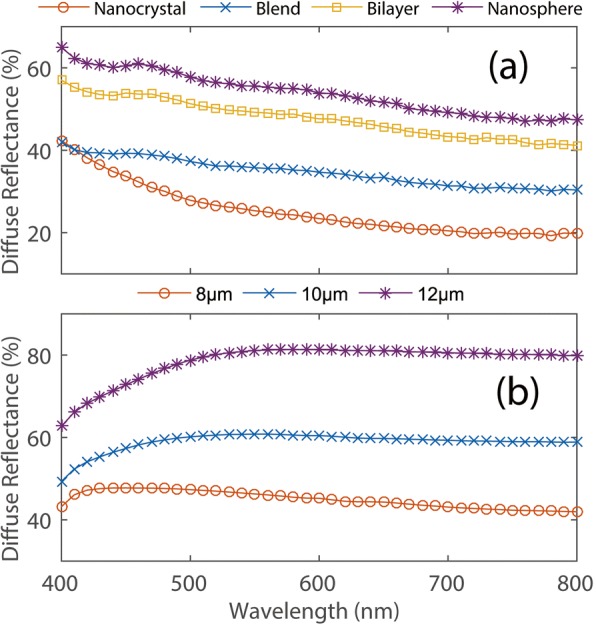
**a**, **b** Diffuse reflectance spectra of the four samples with identical thickness (~ 14 μm) and the optimized nanosphere coatings with different thicknesses, respectively

The diffuse reflectance of the four samples was measured using a spectrophotometer. The measurement wavelength range was 400–800 nm which covers the visible region relevant for the working of display and solar cells. The results obtained are presented in Fig. 2a. From Fig. 2a, it can be seen that blend coating constructed from the mixture of ellipsoidal nanocrystals and spheroidal assemblies exhibits a higher reflectance compared with pure nanocrystal coating. But, even though the ratio of nanocrystals to polymer spheres is approximately the same in these coatings, the diffuse reflectance of the bilayer coating is still higher than that of the blend coating. It suggests that the scattering properties of coatings made by spheroidal assemblies may be better than nanocrystals. Indeed, compared with the other three coatings, nanosphere coating possesses a best scattering effect because the coating is solely constructed by spheroidal assemblies.

Now, it is clear that these simple spheroidal assemblies, which are composed of ellipsoidal TiO_2_ nanocrystals, can be considered as superior scattering particles to enhance the diffuse reflectance. But as shown in Fig. 2a, the average reflectance for nanosphere coating is about 55%, but for certain wavelength ranges (e.g., > 700 nm), the reflectance becomes less than 50%. Moreover, it is worthy to note here that the reflectance value plummets in the visible region, indicating the weak scattering effect of low-energy photons induced by small size of unit cells.

In order to further optimize the diffuse reflectance of the pure spheroidal assembly coating, the sizes of nanocrystals and spheroidal assemblies were increased through adjusting the dosage of precursor. The measured diffuse reflectance spectra corresponding to the optimized nanosphere coatings with enlarged unit cell size and for the different thicknesses (8, 10, and 12 μm) are shown in Fig. 2b. For 8 μm thickness of nanosphere coating, the average reflectance increases above 40% and remains high throughout the whole wavelength range. But as observed in Fig. 2b, the reflectance of nanosphere coating strongly depends on the thickness or, in other words, on the packing fraction of the unit cell. When the thickness of the coating is thin, the packing fraction of ellipsoidal nanocrystals in a spheroidal assembly decreases. Even if the size of the spherical component has been optimized, the hybrid spheroidal structures of thin coatings could not shield the scattering lights properly. And a major portion of the incident light is directly transmitted by the coating. On the other hand, there tend to be more lobes in the scattering diagram near the directions toward which the particle presents a large width than near the directions for which the projected width is smaller [25]. Note that ellipsoidal TiO_2_ nanocrystals oriented with their symmetry axes oblique to the incident beam scatter asymmetrically about the forward direction in Fig. 2b. It means that incident light will be scattered randomly by spheroidal assemblies composed of multi-oriented ellipsoidal TiO_2_ nanocrystals. Thus, it is possible to get a higher diffuse reflectance from the thicker nanosphere coating, because in which the forward scattering may be suppressed by the multi-oriented ellipsoidal TiO_2_ nanocrystals.

### The Structural Details of Hybrid TiO_2_ Nanosphere Coatings

**Fig. 3:**
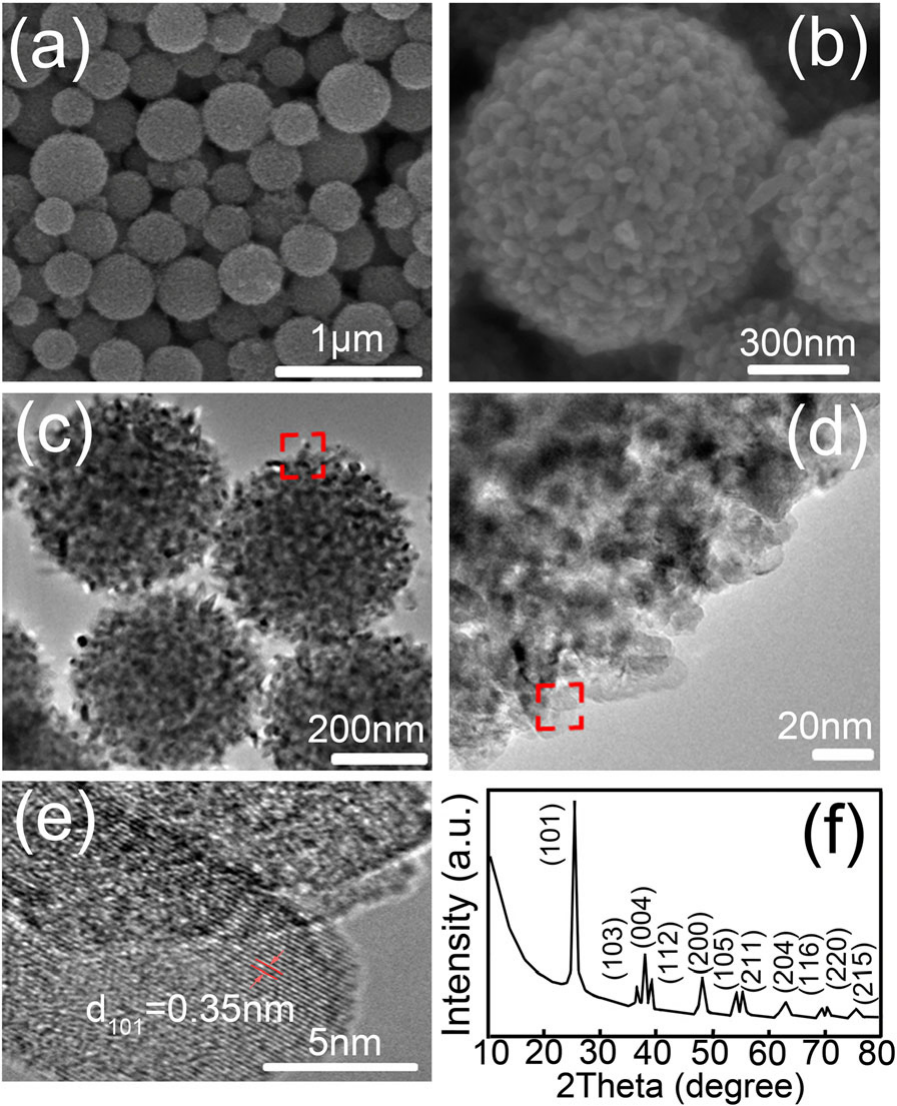
The **a**, **b** SEM, **c**, **d** TEM, and **e** high-resolution TEM images of the nanosphere coating. The **d**, **e** the zoom-in TEM images which gives the details of the area corresponding to the red boxes in (**c**, **d**), respectively. The XRD pattern of the nanosphere coating (**f**)

The information about structural properties of nanosphere coating used in Fig. 2b can be seen clearly in Fig. 3. As depicted in Fig. 3a, the diameter of the spheroidal assembly ranges from 100 to 600 nm, with an average size of 330 nm. In general, for sufficiently large nanospheres (radius of equal volume sphere greater than about 300 nm in visible band), the larger the sphere, the more heavily forward scattering directions are weighted compared with backscattering directions [25]. But as can be seen in Fig. 3b, the enlarged SEM image shows that the nanospheres are assembled from multi-oriented nano-sized ellipsoidal nanocrystals about several nanometers in diameter and several tens of nanometers in length. Compared with the well-defined spheres with uniform diameter, the spheroidal assemblies could increase the backward scattering of the incident light rays and lead to a better diffuse reflection when used as diffuse reflector. In addition, as shown in Fig. 3c, the structural details of these spheroidal assemblies can be obtained by transmission electron microscopy (Tecnai F30). The corresponding TEM image shows that these spheroidal assemblies possess mesoporous structures (Fig. 3c). Moreover, the ellipsoidal nanocrystals at the surface of the sphere exhibit sharp tips and spindle-like configuration (Fig. 3d). As known, the geometry irregularities at surfaces can bring considerable light scattering responses [8, 9, 21]. In fact, using similar TiO_2_ nanospindles as the scattering overlayer in solar cells, efficient light scattering has been observed experimentally [26]. On the other hand, the investigation on variations in layer thickness can be applied to point out some essential differences between surface and bulk processes. It is apparent that bulk scattering increases with layer thickness of nanosphere coating as shown in Fig. 2b, since it depends on the integral in the volume of the stationary zero-order electromagnetic field [10]. Thus, it is possible that both bulk and surface scattering benefit from this nanosphere coating. Furthermore, in the high-resolution TEM image of the tip area of an individual nanospindle (Fig. 3e), the well-defined lattice fringes with inter-plane spacing of 0.35 nm indicate that the primary nanospindles are highly crystallized. Similarly, the XRD pattern of the nanosphere coating suggests the products exhibit well-crystallized structure (Rigaku D/max-2500 diffractometer with Cu Kα radiation, λ = 0.1542 nm, 40 kV, 100 mA), in which all the diffraction peaks can be indexed to anatase TiO_2_ (JCPDS no. 21-1271). It is obvious that the diffraction peaks belonging to (103), (004), and (112) are integrated together, indicating the broadening of the diffraction peaks due to the different particle size.

### The Underlying Scattering Mechanism of Hybrid TiO_2_ Nanosphere Coatings

**Fig. 4:**
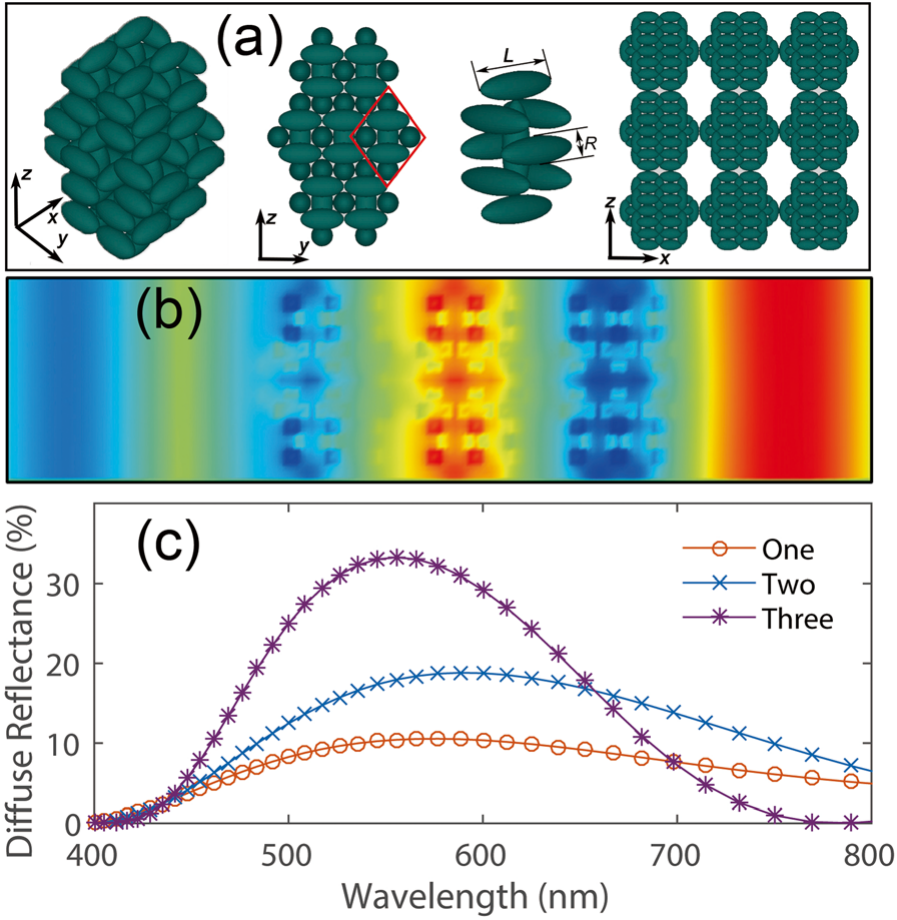
**a** The schematic of the nanosphere assemblies: from left to right, the perspective, front view, the unit cell of the assemblies, and the three layer nanosphere coating utilized in FDTD simulation, respectively. **b** Electric field profile in three layer nanosphere coating. **c** Calculated diffuse reflectance of the nanosphere coating

To explore the nature of these structures, FDTD simulations were conducted using models with geometric size identical to those of the measured samples in experiments by commercial software (East FDTD, Dongjun technology, Shanghai, China). The corresponding model of the nanosphere coating utilized in FDTD simulations is shown in Fig. 4a. The length L and radius R of the ellipsoidal nanocrystal are selected as 60 nm and 30 nm, respectively. And the assemblies (as shown in Fig. 3) are grown through closely packed structure of nanocrystals. In order to simplify the consideration, the different thicknesses of the coating are replaced by changing the layer number of nanospheres. The electric field profile for wavelength 600 nm is shown in Fig. 4b, where light through the coating is scattered uniformly by the coating and resonates inside the assemblies. Thus, we can conclude that, when light is incident from the top side of the nanosphere coating, it gets trapped by the assembly and gradually diverges backward due to the multi-oriented nanocrystals and the scattering effect. In fact, the backward scattering behavior of light in nanosphere coating depends on the amount of spherical assemblies. As can be seen in Fig. 4c, the reflectance of the three-layer nanosphere coating has been significantly improved in the visible wavelength band corresponding to that of the single/two layer(s) coating.

### The Polarization-Dependent Specular Reflectance Properties of Hybrid TiO_2_ Nanosphere Coatings with Different Thicknesses

As well known, reflection spectra of almost all crystal types of titanium dioxide are in the ultraviolet region below 400 nm [27, 28]. Therefore, titanium dioxide frequently appears in many sunscreen cosmetics aiming to reduce the damage of ultraviolet rays to human skin. However, in the visible light region, its efficiency decreases as the transmittance increases. There is great significance on how to improve the reflection efficiency of titanium dioxide in the visible light region.

**Fig. 5:**
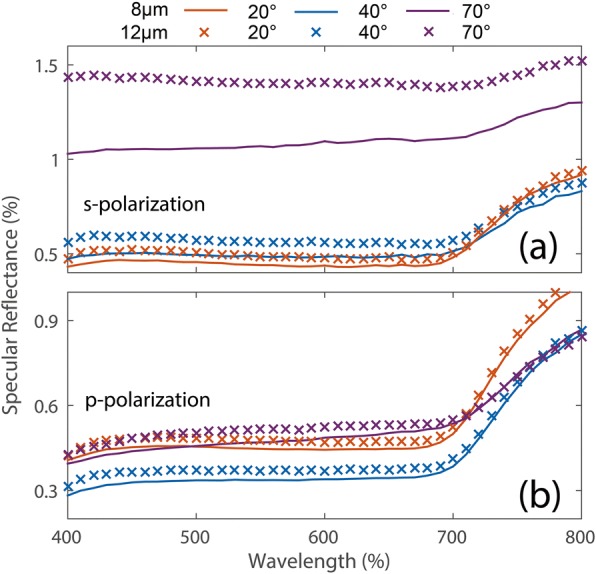
The specular reflectance of the nanosphere coatings with different thickness for s- (**a**) and p- (**b**) polarizations, respectively

We further analyzed the polarization-dependent specular reflectance of the nanosphere coatings using spectrophotometer (Agilent Carry 5000). The obtained results for the optimized nanosphere coatings at two different thicknesses (8 and 12 μm) are shown in Fig. 5. The specular reflectivity of the two samples in the spectral region of 400–700 nm is maintained at a low level (less than 2%), which proves the previous discussion. The results show that the nanosphere coating has a strong ability in suppressing specular electromagnetic wave reflection in the spectral region of 400–700 nm for both normal and wide-angle incidences. However, the specular reflectance of the two samples in the 700–800 nm range has a significant upward trend for different angles and polarizations. This anomalous phenomenon probably comes from the effect of the nanotopography of titanium dioxide. Previously, it has been demonstrated that reflective coatings composed of titanium dioxide with different structural topographies have a great influence on the reflection band. For instance, the light-scattering of titanium dioxide around 400 nm and 700 nm can be improved by adopting different structures, nanorod, nanowire, and nanosphere [29]. Here, our results also prove this point.

In addition, the bandwidth and amplitude of specular reflection reduction are insensitive to the polarization of the incident light and the thickness of the coating. As stated above, these special properties can be attributed to the fact that the sphere assembly is a collection of many randomly oriented particles, which may themselves be anisotropic. However, the results also show that the proper polarization may have an effect on the reflection efficiency of the coating, which provides more possibilities for future designs.

## Conclusions

In conclusion, we report a new technique to enhance diffuse reflectance in a hybrid TiO_2_ microstructured coating. Depending on the shape of the TiO_2_ nanoparticles in the coating, the incident light is reflected uniformly due to the multi-oriented nanocrystals and the scattering effect. These hybrid microstructured coatings are grown through a low-cost solvothermal method by altering the peroxotitanium complex precursor dosage. By increasing the size of the ellipsoidal TiO_2_ nanocrystals, we optimized our structure to achieve a maximum reflectance of approximately 80% over the wavelength range of 550 nm to 800 nm. With the help of fine structure and morphology characterization, we have analyzed the behavior of the measured reflectivity spectrum with the change in thickness and verified the result with FDTD simulation. Finally, a wide-angle, polarization-insensitive specular reflection reduction can be found in these nanosphere coatings. And the maximum specular reflectance at any wavelength is less than 1.5% for the whole broadband (400–800 nm) range of wavelengths. Our proposed hybrid microstructured coatings with its unique light scattering and tunable ability will be useful for highly efficient diffuse reflector or for applications in various advanced fields of photonics related to light extractions and diffusers. There is a further scope of investigations on the effect of the diameter, orientation, and distributions of the ellipsoidal TiO_2_ nanocrystal in the spherical assemblies on the light manipulation mechanism.

## Abbreviation

FDTD: Finite difference time domain
